# Exosomal miR-940 maintains SRC-mediated oncogenic activity in cancer cells: a possible role for exosomal disposal of tumor suppressor miRNAs

**DOI:** 10.18632/oncotarget.15525

**Published:** 2017-02-20

**Authors:** Mohammed H Rashed, Pinar Kanlikilicer, Cristian Rodriguez-Aguayo, Martin Pichler, Recep Bayraktar, Emine Bayraktar, Cristina Ivan, Justyna Filant, Andreia Silva, Burcu Aslan, Merve Denizli, Rahul Mitra, Bulent Ozpolat, George A. Calin, Anil K. Sood, Mohamed F. Abd-Ellah, Gouda K. Helal, Gabriel Lopez Berestein

**Affiliations:** ^1^ Department of Experimental Therapeutics, The University of Texas MD Anderson Cancer Center, Houston, TX, USA; ^2^ Department of Center for RNAi and Non-Coding RNA, The University of Texas MD Anderson Cancer Center, Houston, TX, USA; ^3^ Department of Gynecologic Oncology and Reproductive Medicine, The University of Texas MD Anderson Cancer Center, Houston, TX, USA; ^4^ Department of Pharmacology and Toxicology, Faculty of Pharmacy, Al-Azhar University, Cairo, Egypt; ^5^ Instituto de Investigação em Saúde, Universidade do Porto, Porto, Portugal; ^6^ INEB-Institute of Biomedical Engineering, Universidade do Porto, Porto, Portugal; ^7^ Division of Clinical Oncology, Department of Medicine, Medical University of Graz, Graz, Austria

**Keywords:** exosomes, ovarian cancer, miR-940, SRC, tumor suppressive

## Abstract

Exosomes have emerged as important mediators of diverse biological functions including tumor suppression, tumor progression, invasion, immune escape and cell-to-cell communication, through the release of molecules such as mRNAs, miRNAs, and proteins. Here, we identified differentially expressed exosomal miRNAs between normal epithelial ovarian cell line and both resistant and sensitive ovarian cancer (OC) cell lines. We found miR-940 as abundant in exosomes from SKOV3-IP1, HeyA8, and HeyA8-MDR cells. The high expression of miR-940 is associated with better survival in patients with ovarian serous cystadenocarcinoma. Ectopic expression of miR-940 inhibited proliferation, colony formation, invasion, and migration and triggered G0/G1 cell cycle arrest and apoptosis in OC cells. Overexpression of miR-940 also inhibited tumor cell growth *in vivo*. We showed that proto-oncogene tyrosine-protein kinase (SRC) is directly targeted by miR-940 and that miR-940 inhibited SRC expression at mRNA and protein levels. Following this inhibition, the expression of proteins downstream of SRC, such as FAK, paxillin and Akt was also reduced. Collectively, our results suggest that OC cells secrete the tumor-suppressive miR-940 into the extracellular environment via exosomes, to maintain their invasiveness and tumorigenic phenotype.

## INTRODUCTION

Ovarian cancer (OC) is one of the leading causes of death among women with gynecologic malignancies. According to the National Cancer Institute, approximately 21,290 new cases and 14,180 deaths were reported in 2015 [1]. Unfortunately, the majority of cases are diagnosed at an advanced stage, and an overall 5-year survival rate of approximately 40%. At present, the mainstay of care for newly diagnosed OC is surgical debulking followed by adjuvant chemotherapy (mainly taxanes and platinum compounds) [2, 3]. Most patients with OC relapse, highlighting the urgent need for new treatment strategies for the elimination of OC. Up to date, strategies targeting only tumor cells have proven to be unsuccessful to eliminate OC and prevent metastasis. Therefore understanding tumor and tumor microenvironment and identification of novel “molecular targets/ pathways” required for the development of the highly effective therapies to eradicate OC and improve patient survival.

Exosomes are small membrane-derived vesicles of about 30-140 nm that are thought to be shed from a variety of cell types [4]. Cancer cells have been shown to secrete exosomes in greater amounts than normal cells [5]. They can also be found in various body fluids under both healthy and morbid conditions [6, 7]. Exosomes may carry various cargos from their donor cells, including membrane receptors, specific proteins and nucleic acids (DNA, mRNAs, miRNAs, and other non-coding RNAs), that can be translated into functions in the recipient cells [8]. The vesicles were initially proposed as a mechanism through which cells discard transferrin receptor during the maturation of reticulocytes [9].

MicroRNAs (miRNAs) are endogenous small, noncoding RNAs that act as post-transcriptional regulators of gene expression through the degradation of target mRNA or through translational inhibition [10]. Several studies have shown that miRNAs are involved in cell proliferation, intercellular signaling, cellular differentiation, apoptosis, and immune modulation [11, 12]. miRNAs are aberrantly expressed in cancers, indicating that they may function as oncogenes or tumor suppressors in signaling pathways involved in cancer initiation and progression and in the development of metastasis [13, 14]. Dysregulation of miRNA expression levels could be attributed to several mechanisms, including genomic deletion, mutation, or their active secretion as membrane-bound vesicular content and transfer to recipient cells [15, 16]. For instance, tumor-suppressive miRNAs in the exosomes from normal epithelial cells inhibited the proliferation of cancer cells not only *in vitro* but also *in vivo* [17].

Several reports have shown that circulating miRNAs are associated with OC and correlate with disease detection, severity, and response to treatment [18–22]., but the function of these exosomal miRNAs remains elusive and poorly understood. Therefore, identifying the oncogenic and tumor suppressor exosomal miRNAs is an important step toward developing new strategies for both the diagnosis and treatment of OC.

In the present study, we hypothesized that the release of miRNAs from OC cells into extracellular fluids via exosomes is a selective process, and the relative abundance of tumor-suppressive miRNAs are higher in exosomes from OC cells compared with their cellular expression or exosomes derived from normal ovarian cells. We also hypothesized that the secretion of the suppressor miRNAs by cancer cells results in depletion of these miRNAs and intracellular activation of oncogenic pathways. In this study, we selected miR-940 since we observed that its expression was higher in three different ovarian cancer cell exosomes compared to normal epithelial ovarian cell exosomes.

## RESULTS

### Exosome isolation and characterization

Initially, for the purpose of profiling exosomal miRNAs, we first isolated exosomes from culture media of six OC cell lines after 24 hours of incubation using total exosome isolation reagent as described in Materials and Methods. Previously, the most common method for isolating exosomes from cultured cell media was differential centrifugation, which is very time consuming and requires extensive training to ensure successful isolation of exosomes. Although polymer-based exosome extraction technologies may co-precipitate other proteins and vesicles, we selected a commercial reagent as a translatable means of obtaining enriched exosome-derived RNA from small-volume samples, an approach validated by other researchers [23–25]. To confirm the efficiency of the isolation method and the quality of the vesicles, we followed an extensive characterization.

**Figure 1:**
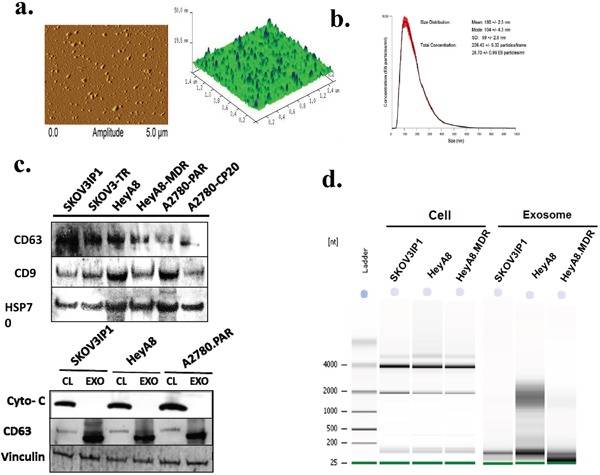
Characterization of exosomes and exosomal miRNA isolated from ovarian cancer cells **a**. Atomic force microscopy images of exosomes showing the morphology and size distribution of vesicles. Exosomes appeared as isolated vesicles with characteristic round-shaped structures in a 3D topographic image. **b**. Nanoparticle tracking analysis (NTA) of SKOV3IP1 exosomes. The graph represents the size distribution of particles in solution showing an average of the mode size of 104 nm. **c**. Upper panel: Western blot analysis of ovarian cancer derived exosomes. Exosomal marker proteins CD63, CD9, and HSP70 were detected in exosome preparations. Lower panel: Cytochrome C (Cyto-c) was detected in cell lysates (CL) but was not detectable in exosomes (EXO), which may indicate that the exosome preparations were not contaminated by apoptotic body vesicles. CD63 and vinculin are used as loading controls. Each experiment was replicated 3 times and representative blots are depicted. **d**. Exosome and cellular RNA were analyzed using the Agilent 2100. Gel obtained with Agilent 2100 Bioanalyzer showing the relative increase in the exosomes of small RNAs (below 200 nucleotides), including miRNAs, but no or very low amount of ribosomal RNA (18S- and 28S- rRNA) compared to their donor cells.

We assessed the morphology and size using Atomic Force Microscopy (AFM), which showed that the isolated exosomes appeared as vesicles with characteristic circular structures in 3D topography (Figure 1a). We analyzed ~320 vesicles and found that the mean size of OC-derived exosomes was 51.01 nm (Supplementary Figure 1a). This size is consistent with previously reported characteristics of exosomes [15, 26]. Since the characteristic shape and size of exosomes are distinct from any other structures seen on the surface, the height profile of 3 individual exosomes and the size distribution of exosomes are shown in Supplementary Figure 1b, which shows near homogeneity with respect to height and width.

Because AFM examines only pelleted or solid surface-bound vesicles, we next selected Nanoparticle Tracking Analysis (NTA), which is suitable for studying particle size in suspension. The NTA for SKOV3ip1 revealed an average mode value of 104 ± 4.3 nm (Figure 1b).

We further evaluated by Western blotting the expression of several exosome markers in proteins isolated from all six OC cell lines. Three well-known exosomal markers, CD63, CD9, and Hsp70, were found to be present in all OC-derived exosomes [4, 27]. (Figure 1c, upper panel). Cytochrome c, a mitochondrial protein, was detectable in whole-cell lysates but absent in the exosomes, indicating that the exosome preparations were not contaminated with apoptotic vesicles (Figure 1c, lower panel). CD63 was used as a control for exosome expression. Together, these findings verified that the examined vesicles were exosomes and could be isolated in a consistent manner.

### MicroRNA profiles of exosomes and their parental cells and validation by qRT-PCR

**Figure 2:**
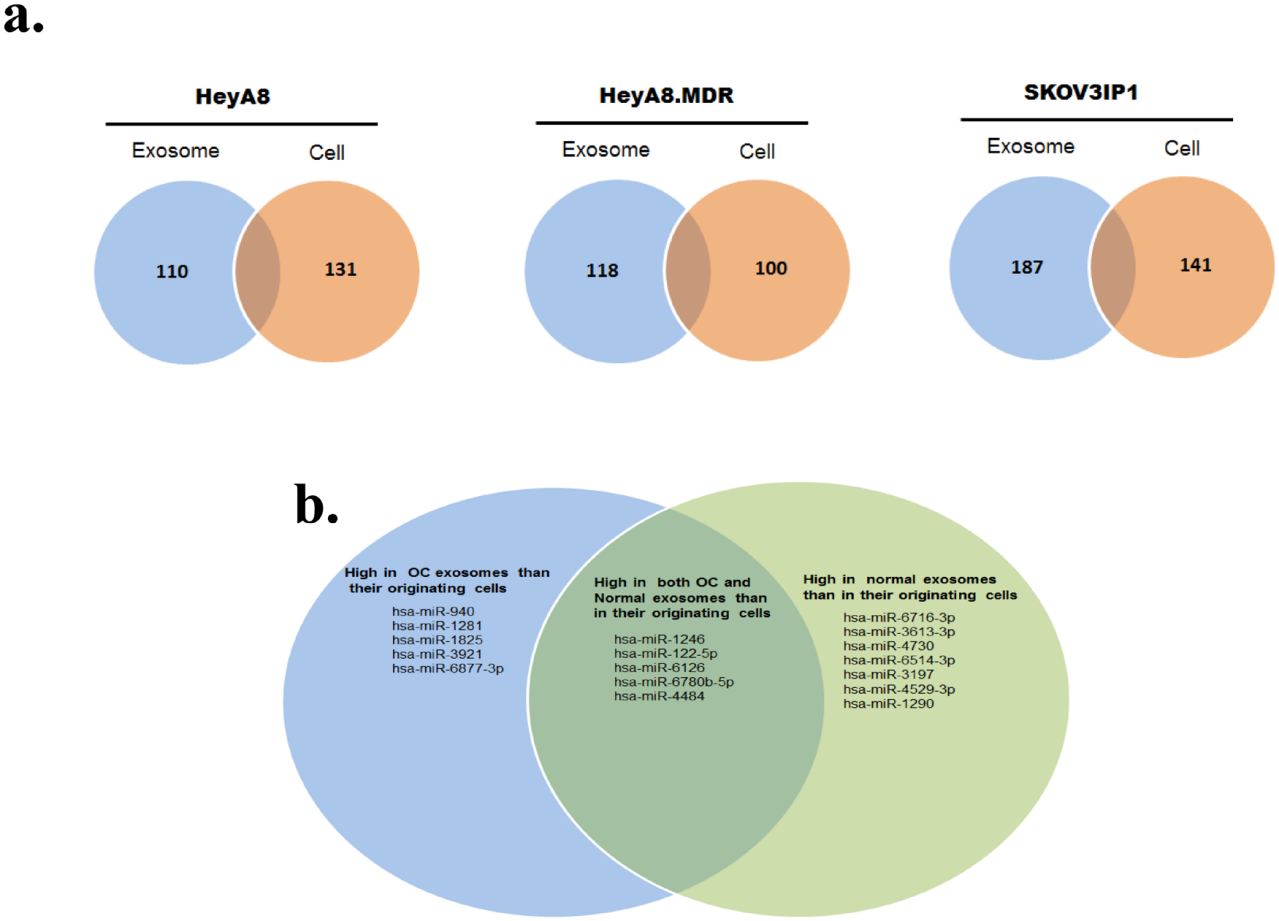
Profiling of miRNAs isolated from OC cells and their corresponding exosomes **a**. Venn diagrams based on miRNA microarray results showing the miRNAs most enriched in exosomes or in their respective donor cells. **b**. Venn diagram showing the miRNAs highly enriched in OC-derived exosomes versus normal exosomes and vice versa.

To confirm whether RNA was properly conserved in exosome samples, we examined total RNA isolated from OC exosomes and their cells of origin using an Agilent Bioanalyzer 2100. Consistent with previous studies of exosomes from other cell types [28–30], the exosomal RNA showed little or no peaks of 28S and 18S ribosomal RNA compared to cellular RNA, whereas the characteristic peak of small RNA was detected below 200 nucleotides (Figure 2a and Supplementary Figure 2b).

Next, we evaluated miRNAs differentially expressed in exosomes versus their cells of origin, using the Affymetrix GeneChip miRNA 4.0 microarrays, which contain 36,353 unique probes based on miRBase v20. After the normalization of each miRNA across all samples, one-way ANOVA was performed to identify significantly expressed mature miRNAs. To identify the most prominent miRNAs that were differentially expressed in exosomes versus cells, we restricted the criteria for upregulation as ANOVA corrected *P* <0.05 and a fold change of ≥ 5 and the criteria for down-regulation as ANOVA corrected *P* <0.05 and a fold change of ≤−5.

Microarray analysis showed that certain miRNAs were expressed at higher levels in exosomes than in their donor cells and vice versa (Figure 2a and Additional File 1), which may suggest cell type-specific mechanisms for highly selective enrichment or exclusion of the miRNA in exosome-mediated secretion. Several miRNAs (miR-940, miR-1281, miR-1825, miR-3921, and miR-6877-3p) were significantly more abundant in exosomal samples from SKOV3IP1, HeyA8, and HeyA8-MDR OC cells, whereas other miRNAs (miR-1246, miR-122-5p, miR-6126, miR-6780b-5p, and miR-4484) were more abundant in both OC and normal exosomes than in their originating cells (Figure 2b).

To identify the potential roles of exosome-derived miRNAs, we focused on the 5 miRNAs that were highly enriched in OC-derived exosomes and not elevated in normal exosomes. We excluded miR-6780b-3p, since it is a new miRNA and has no predicted targets by most target prediction algorithms. As an initial step toward understanding the function of the preferentially exported miRNAs, we attempted to determine the genes being targeted by these miRNAs. We employed DIANA microT-CDS, which has been shown to perform well in terms of sensitivity and specificity compared to other available algorithms [31].

**Figure 3:**
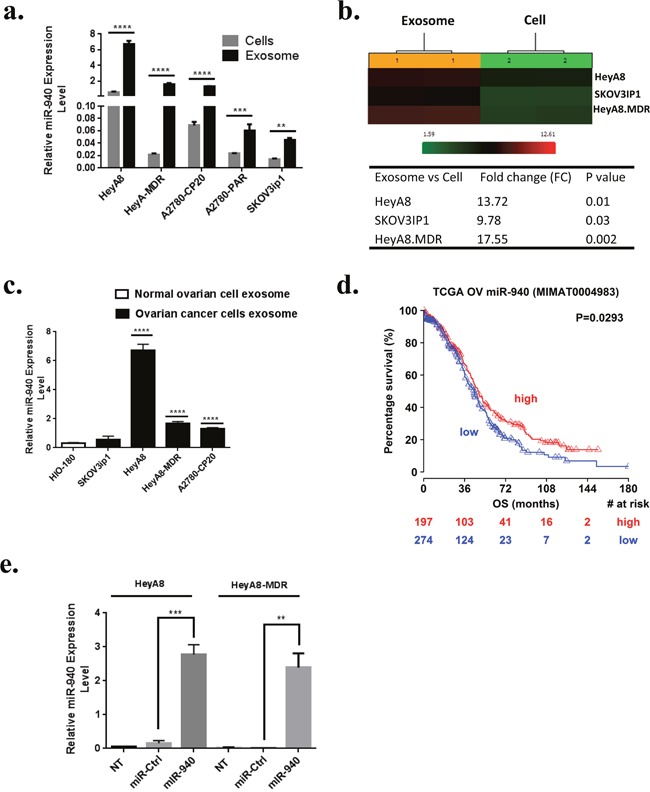
Relative expression of miR-940 in ovarian cells and their exosomes **a** and **b**. Relative expression of miR-940 shows significant up-regulation in exosomes compared to their originating cancer cells (*P* values obtained with Student's *t*-test; **P <0.01; ***P <0.001 or ****P <0.0001; bars and error bars represent mean values and the corresponding standard error (SE)). **c**. Expression levels of miR-940 are significantly higher in OC cells derived exosomes than exosomes derived from HIO180 non-transformed ovarian cell line (****P <0.0001). **d**. Kaplan-Meier plots for miR-940 expression in ovarian cancer patients (The Cancer Genome Atlas data). High expression of miR-940 is associated with better survival. **e**. Ectopic expression of miR-940 in OC cell lines HeyA8 and HeyA8-MDR were evidence by qRT-PCR after transfection.

The genes predicted to be targeted by one or more of the most selectively exported miRNAs are listed in Additional File 3. Functional analysis of these genes revealed that the most enriched pathway was adherens junction (hsa04520) (*P* = 1.48E-09), which contained 20 predicted target genes (Supplementary Figure 3). All the enriched pathways are listed in Additional File 4. We focused in particular on miR-940 for further studies as it is novel and has never previously been associated with OC. Next, we examined the intracellular and extracellular levels of miR-940 in different OC cells by real time PCR. The expression of miR-940 was higher at the secretory level than at the endogenous level (Figure 3a). The outcomes were consistent with the normalized microarray data (Figure 3b). Interestingly, microRNA expression levels were examined in OC and normal exosomes, miR-940 was also significantly higher in OC exosomes than in normal exosomes (Figure 3c), which is in line with the microarray data (Additional File 2). We, then analyzed the effect of miR-940 in ovarian cancer patient survival by Kaplan-Meier plots. The Cancer Genome Atlas (TCGA) data revealed that high expression of miR-940 is associated with better survival in patients with ovarian serous cystadenocarcinoma (Figure 3d).

### MicroRNA-940 overexpression suppresses OC cell proliferation and colony formation and induces G0/G1 cell cycle arrest

**Figure 4:**
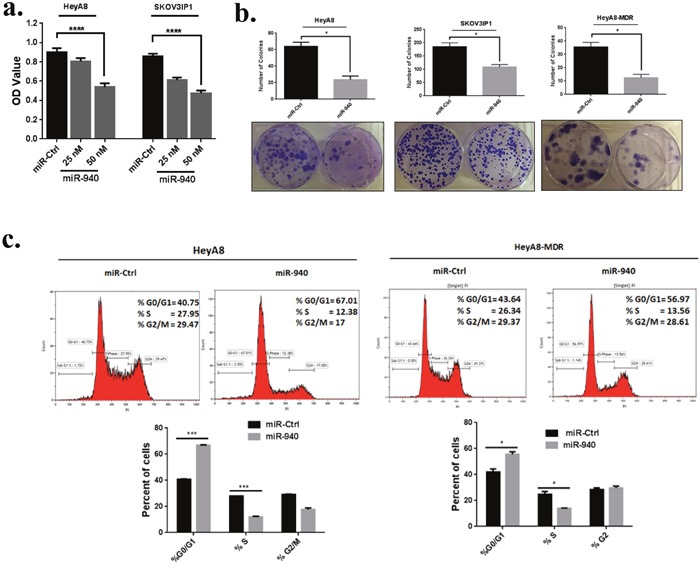
miR-940 inhibits OC cell growth *in vitro* and induces G0/G1 cell cycle arrest **a**. Overexpression of miR-940 significantly inhibited HeyA8 and SKOV3IP1 cells viability. Cells were transfected with indicated miRNAs, and after 72 h, Cell growth rates were detected by MTS assay. Data are represented as mean ± SE. ****P <0.0001 versus control cells from three independent experiments. **b**. Ectopic expression of miR-940 significantly inhibited colony formation in HeyA8, HeyA8-MDR and SKOV3IP1 cells (*P < 0.05 indicate significant difference compared with control group). The results are presented as means ± SE (*n* = 3 for each panel). **c**. Cell-cycle arrest analysis of HeyA8 and HeyA8-MDR cells after 72 h transfection with miR-940. Cells were harvested at 72 h and were fixed, stained with propidium iodide (PI) and analyzed by fluorescence-activated cell sorting. Data are presented as the percentage of cells as mean ± SE. *P <0.05, ***P <0.001 (Student's t-test). Two independent experiments were performed and the representative one was shown.

Because the expression of miR-940 was significantly lower in OC cell lines than in their exosomes, we next focused on the functional effects of miR-940 on OC cells. MiR-940 and miR control were transiently transfected into HeyA8 and HeyA8-MDR cells. qRT-PCR analysis showed that miR-940 was remarkably (~1.7×10^3^ fold and ~14×10^3^ fold) increased in HeyA8 and HeyA8-MDR cells, respectively transfected with miR-940 mimics compared to miR-mimics control (Figure 3d). To determine whether overexpression of miR-940 has an effect on cell growth in OC, we performed a proliferation assay in OC cell lines. The results of the MTS assay showed that miR-940 overexpression significantly reduced the growth rates of both HeyA8 and SKOV3IP1 cells, by 40% (*p < 0.0001*) and 45%, respectively (*p < 0.0001*) (Figure 4a). Colony formation assays further confirmed the anti-proliferative function of miR-940 in OC cells (Figure 4b). Concomitant with this inhibition of cell proliferation, there was a significant cell cycle arrest in the G1 phase, which was evidenced by an approximately 63% and 33% increase in the percentage of HeyA8 and HeyA8-MDR cells, respectively accumulating in the G0/G1 phase, accompanied with a decrease in the S phase population in both cell lines (Figure 4c).

### Ectopic expression of miR-940 induces cell apoptosis by regulating caspase-dependent apoptosis pathways

**Figure 5:**
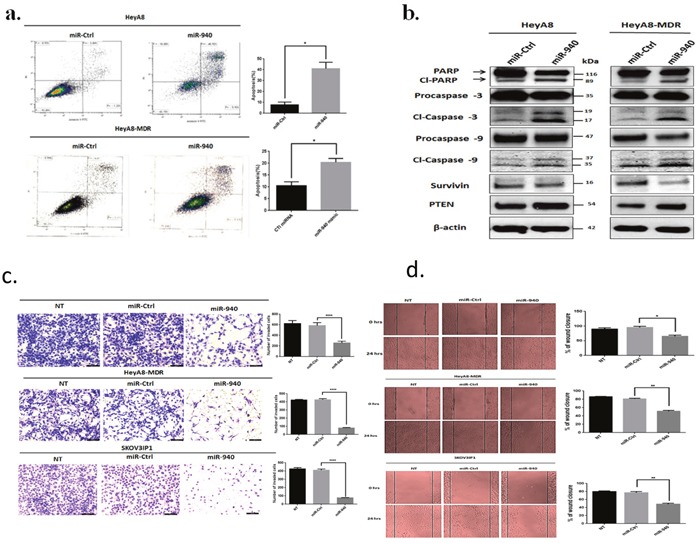
Transient transfection of miR-940 triggers apoptosis and suppresses the invasiveness and migration of OC cells **a**. Ectopic expression of miR-940 triggered apoptosis in HeyA8 and HeyA8-MDR cells. Cells were transfected with miR-940 or control mimic, and analyzed by Annexin V-FITC and PI double-staining and positive cells were detected and quantified by FACS analysis. The represented percentages show positive cells at both early and late apoptosis. Data are represented as mean ± SE. * P < 0.05 indicates significant difference compared with control group. All experiments were independently performed three times. **b**. Apoptotic-related proteins expression was detected by western blot. miR-940 induced apoptosis was manifested after 72 h of transfection, by increase in cleavage of Caspase-3, 9 and PARP as well as a marked decrease in anti-apoptotic Survivin with a concomitant increase in PTEN. β-actin was used as a loading control. **c**. Invasion assay showed a significant decrease in the number of invading HeyA8, HeyA8-MDR and SKOV3IP1 cells transfected with miR-940. Cells were transfected with indicated miRNAs and seeded onto Matrigel-coated Transwell filters in Matrigel invasion chambers. The invaded cells in a minimum of four random selected fields were counted and data are presented as mean ± SE. Each experiment was performed at least in triplicate. ****P <0.0001 indicates significant difference versus control. **d**. Wound healing assay showed that the migration of OC cells was significantly decreased after transfection of miR-940 mimics compared with control groups. A single scratch was made in the center of the confluent cell monolayer, and the wounded monolayers were transfected with indicated miRNAs. The migration distance was measured at 0 and 24 h after the cells had been scratched. The data is expressed as mean of the percentages of wound closures ± SE of three independent experiments. (* P < 0.05; **P <0.01 represent significant difference between indicated groups.

Inhibition of cell growth in cancer cells is usually associated with concomitant activation of cell death pathways. We therefore examined the contribution of apoptosis to growth inhibition mediated by miR-940 overexpression. We evaluated the rate of cellular apoptosis using Annexin V and PI staining for flow cytometry. The number of both early and late apoptotic HeyA8 and HeyA8-MDR cells at 72 hours post-transfection of miR-940 was substantially higher by ~5-fold and~2-fold, respectively than the number of control miRNA transfected cells (Figure 5a). The induction of apoptosis was further confirmed by the expression of apoptosis-related proteins including PARP, caspase-3, caspase-9, survivin, and PTEN on Western blot (Figure 5b).

### MicroRNA-940 inhibits the invasiveness and migration of OC cells

To further determine the effects of miR-940 on malignant phenotypes of OC cells, we assessed whether miR-940 could alter the invasion and migration of OC cells by performing an *in vitro* matrigel invasion assay and wound-healing assay in HeyA8, HeyA8-MDR, and SKOV3IP1 cells. These assays mimic the *in vivo* migration and invasion process [32, 33]. Ectopic overexpression of miR-940 significantly reduced the number of invading HeyA8, HeyA8-MDR, and SKOV3IP1cells by about 56%, 80% and 80%, respectively in comparison with miR-control (Figure 5c). Furthermore, the wound-healing assay demonstrated that transient overexpression of miR-940 significantly decreased the ability of OC cells to migrate (Figure 5d). Compared with the negative control miRNA, miR-940 expression reduced HeyA8, HeyA8-MDR, and SKOV3IP1 cells migration by 31%, 36% and 37%, respectively. These results indicate that miR-940 strongly inhibits OC cell invasion and metastasis potential.

### MicroRNA-940 directly represses proto-oncogene SRC kinase and its downstream genes involved in proliferation, invasion and migration pathways in OC

To investigate the underlying molecular mechanisms of miR-940 in OC growth and metastasis, we searched for the putative target genes of miR-940 using online prediction tools, such as TargetScan [34] and DIANA microT [35]. Because miR-940 was able to inhibit OC proliferation and invasion, we focused on the genes that promoted tumor progression and metastasis. Among the hundreds of different targets predicted, SRC was notable because it is overexpressed in a number of malignancies, such as colorectal cancer, lung cancer, and pancreatic cancer [36–38]. Furthermore, our previous studies demonstrated that SRC expression was markedly up-regulated in OC and involved in a variety of cellular functions, including cell proliferation, invasion, and migration [39–41].

**Figure 6:**
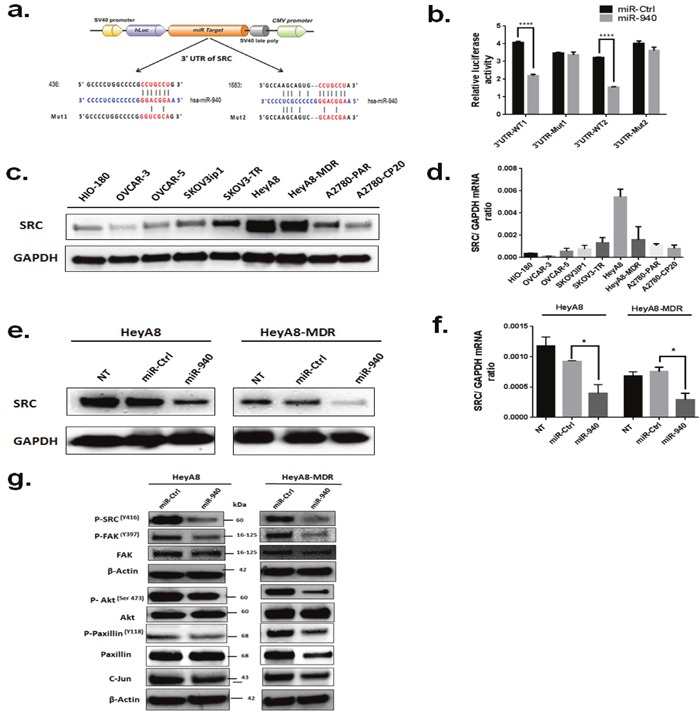
Proto-oncogene SRC kinase is a direct target of miR-940 **a**. Sequence alignment of the wild-type (WT) and mutant (MUT) SRC 3′-UTR, indicating the potential binding sites for miR-940. The seed regions of miR-940 and the seed-recognizing sites in the SRC 3′-UTR are indicated in red. **b**. Luciferase reporter assay of HEK-293 cells which were co-transfected with the luciferase reporter plasmid containing the wild-type (WT) or mutant (MUT) 3′UTR of SRC gene and 50 nM of either miR-940 mimic or control mimic. Luciferase activity was determined using the dual luciferase assay and shown as the relative firefly activity normalized to renilla activity. Significant differences was found between scramble and miR-940 on wild-type 3′UTR construct but not with construct carrying a mutated seed region. The data are shown as mean±. SE of three separate experiments. ****P <0.0001indicates significant difference compared with control group. **c**. Expression levels of SRC in different ovarian cell lines detected by western blot. Levels of GAPDH were evaluated as an internal control for loading. **d**. Expression levels of SRC mRNA in ovarian cancer cells detected by real-time PCR. The mRNA expression levels were normalized against GAPDH. **e**. Measurement of SRC protein expression levels in HeyA8 and HeyA8. MDR cells by western blot analysis following transfection with the miR-940 mimic or miR-Ctl. β-actin was used as an internal control. **f**. Measurement of SRC mRNA expression levels in HeyA8 and HeyA8. MDR cells by RT-qPCR following transfection with the miR-940 mimic or miR-Ctl. GAPDH was used as an internal control. **g**. Expression of the downstream targets of SRC upon transfection of miR-Ctl or miR-940 in HeyA8 and HeyA8. MDR cells. As shown miR-940 significantly regulates SRC and its downstream genes. Cells were transfected with indicated miRNAs, and cell lysates were subjected to Western blot analysis. β-actin was used as a loading control. Three independent experiments were performed with similar results, and representative data are shown.

Using the target prediction databases, 2 potential binding sites of the 3′-UTR of SRC were predicted (Figure 6a). To assess the regulation of these 2 predicted binding sites, we constructed luciferase reporter plasmids containing SRC 3′UTR sequences (3′UTR-WT1 and 3′UTR-WT2) or with mutated putative miR-940 target sites (3′UTR-Mut1 and 3′UTR-Mut2). After transient co-transfection of HEK293 cells with WT-SRC plasmids, the luciferase activity of miR-940 transfected cells was significantly lower than that of control miRNA transfected cells, while the inhibitory effect of miR-940 on luciferase activity was abolished in mutant construct, Mut-1 and Mut-2(Figure 6b).

Next, we measured protein expression levels of SRC (60 kDa) by Western blot analysis in different OC cell lines and in HIO-180 non-transformed ovarian epithelial cells (Figure 6c). SRC protein was highly expressed in HeyA8 and HeyA8-MDR cells, but its expression was lower in HIO-180 cells. SRC mRNA basal levels were higher in most of the OC cell lines tested than in HIO-180 cells (Figure 6d). The results indicated that the endogenous level of SRC expression was directly associated with that of miR-940 in exosomes.

Because of these observations, we sought to determine whether the tumor suppressor function of miR-940 in OC was attributable to its suppressive effect on SRC expression. Thus, we examined the effect of miR-940 on the endogenous SRC expression level via qRT-PCR and Western blotting in HeyA8 and HeyA8-MDR cells. Transfection with the miR-940 mimic led to an obvious down-regulation of SRC protein and mRNA expression levels in both HeyA8 and HeyA8-MDR cells (Figure 6e, 6f, respectively). Western blot analysis showed reduced levels of SRC downstream molecules that are involved in cell proliferation, migration, and invasion following miR-940 overexpression (Figure 6g). We also determined the effect of miR-940 overexpression in normal ovarian cells (HIO-180) by wound healing assay (Supplementary Figure 4). miR-940 treatment also decreased the migration ability of HIO-180 cells.

Furthermore, we performed rescue experiments using SRC lentiviral vector to overexpress SRC protein in ovarian cancer cells. Over expression of SRC was analyzed by westernblot (Supplementary Figure 5a). Ectopic expression of miR-940 rescued the increased invasive and migratory effect of SRC in ovarian cancer cells. Transfecting cells with miR-940 mimic decrease the migration ability of cells compared to CTL miRNa transfected cells. As expected, the effect of miR-940 treatment on the open wound area in CTL lentiviral transfected cells is smaller than that in SRC lentiviral transfected cells (Supplementary Figure 5b). We observed the same patter in invasion assay (Supplementary Figure 5c). However, we did not observe significant decrease in apoptotic protein levels; such as Caspase-3 and PARP (Supplementary Figure 5d).

The ability to modulate SRC protein and its downstream genes levels might explain, at least in part, why miR-940 can inhibit cell proliferation and invasion in OC. Moreover, the amount of exosomal miR-940 was much greater in HeyA8 and HeyA8-MDR cells than in the other OC cell lines and normal ovarian HIO-180 cells. Interestingly, the endogenous levels of miR-940 target SRC were higher in HeyA8 and HeyA8-MDR cells than in other cells, suggesting that both OC cell lines selectively and actively secreted miR-940 into the extracellular environment via exosomes to maintain the activity of SRC and its downstream genes that are involved in tumorigenesis.

We also used combination treatment to test cancer cell invasion *in vitro*. We found that combination treatment resulted in synergistic effect on invasiveness of ovarian cancer cells (Supplementary Figure 6). miR-940 treatment in combination with SRC siRNA led to significantly lower number of invaded cells compared to SRC siRNA alone (p <0.05).

### Systemic delivery of miR-940 by DOPC nanoparticles suppresses ovarian tumor growth *in vivo*

**Figure 7:**
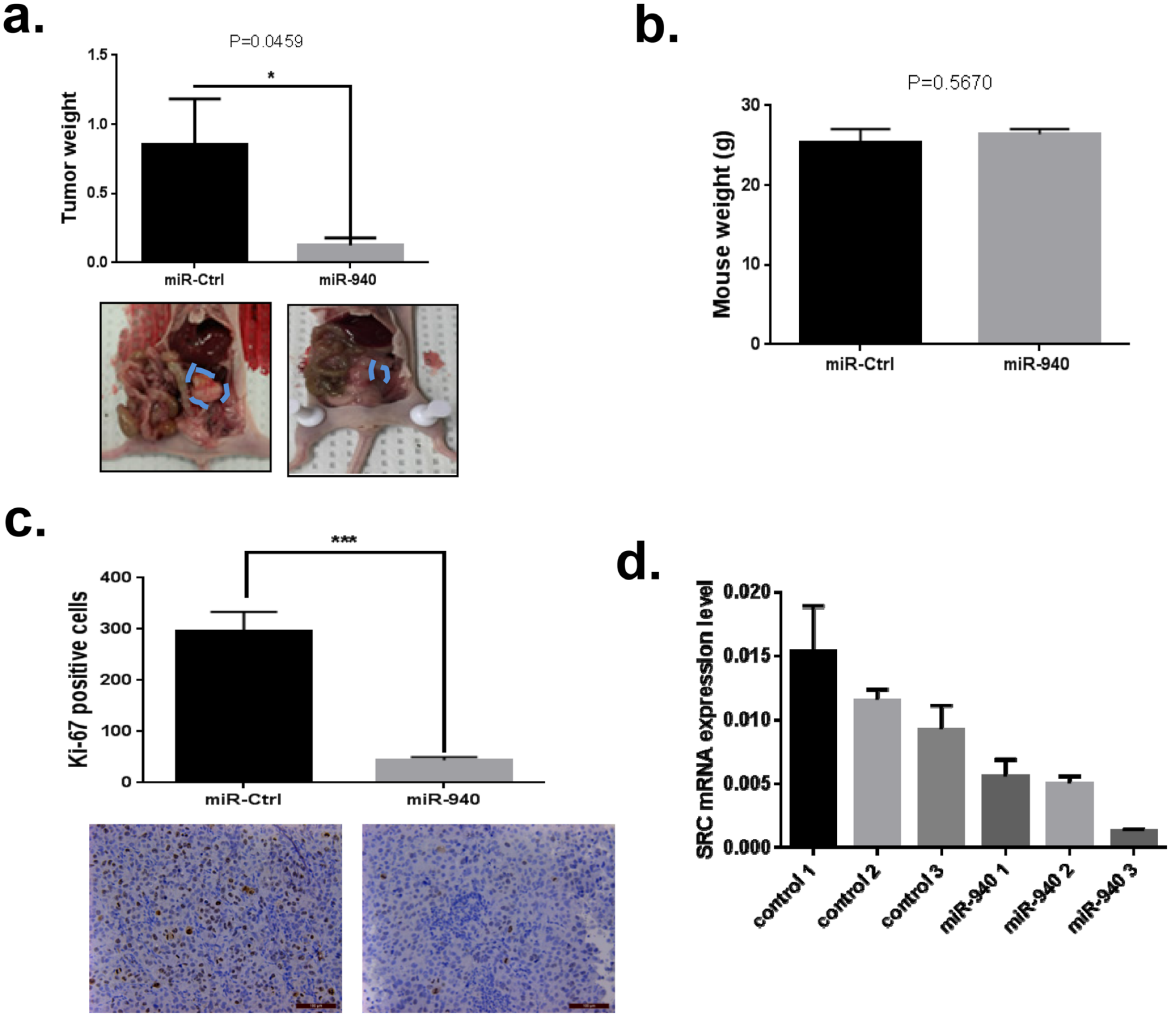
Overexpression of miRNA-940 significantly inhibits tumor growth *in vivo* **a**. The tumor weight of the miR-940 group (g) measured at the end of the experiment were significantly decreased compared to the control group (*P*-values obtained with Student's *t*-test; *P <0.05; compared with control miRNA treated group; bars and error bars represent mean values and the corresponding SE. **b**. Body weight in HeyA8 orthotopic mouse model of OC. No unwanted effects in terms of change in mouse body weight at the end of animal study. **c**. Immunohistochemical staining for tumor proliferation (Ki67) in the HeyA8 orthotopic murine model of ovarian cancer. Quantification of Ki67 positive cells in Control miRNA and miR-940 treated groups were determined from 10 randomly selected fields in a single sample. Two independent reviewers were blinded to the grouping when measuring the percentages of Ki-67-positive cells. The data are shown as mean± SE (n = 10; ***P <0.001 (Student's t-test).vs. control group. **d**. Src protein expression were analyzed using protein lysates obtained from tumors. Src protein levels were lower in groups treated with miR-940 compared to CTL-miRNA.

On the basis of our *in vitro* findings, we next sought to determine whether miR-940 delivery affected tumor growth and whether SRC targets could be down regulated by its delivery *in vivo*. For these studies, we established OC orthotopic mouse models by intraperitoneally injecting mice with HeyA8 cells and treating them with miR-940-DOPC or miR-Ctrl-DOPC. We found that tumors removed from mice treated with miR-940-DOPC were significantly smaller and weighed 85% less (p=0.0459) than those of mice treated with miR-Ctrl-DOPC (*P <0.005) (Figure 7a). No toxicity was observed for any treatment group as indicated by no change in mouse body weight (Figure 7b). Importantly, the isolated tumor tissues were subjected to immunohistochemistry for Ki67 staining to examine the biological effects of miR-940 on tumor cell proliferation [42]. Tumor of mice treated with miR-940 mimic showed 85% reduction in cell proliferation as compared with control group (Figure 7c). These *in vivo* findings were consistent with the *in vitro* results observed in the cell proliferation assays, demonstrating that miR-940 has a tumor-suppressive function.

We, next, examined SRC expression levels in tumors by qPCR (Figure 7d). SRC mRNA levels were significantly decreased in miR-940 treatment groups compared to control group.

### SRC is involved in miR-940-mediated suppression of OC cells tumorgenesis

**Figure 8:**
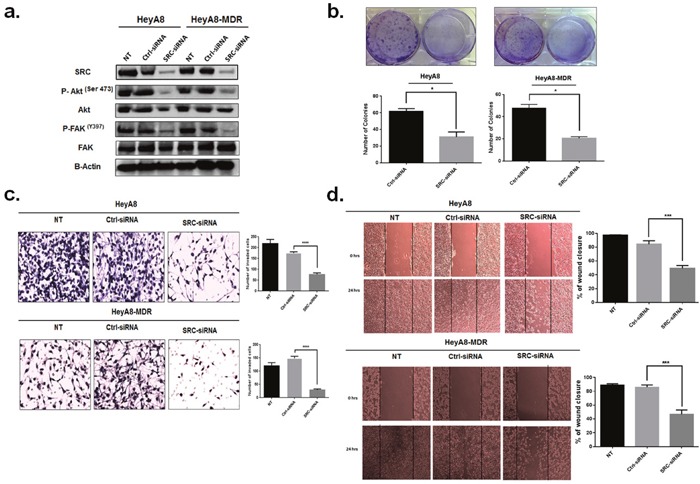
Knockdown of SRC inhibited OC cell proliferation, migration and invasion *in vitro* **a**. Expression of SRC and its downstream genes in HeyA8 andHeyA8. MDR cells transfected with SRC-siRNA or control siRNA (Ctl-siRNA) was detected by Western blot. **b**. Colony formation assay was used to detect the effects on cell proliferation of HeyA8 and HeyA. MDR cells transfected with SRC siRNA. Downregulation of SRC decreased colony formation ability (* p <0.05). The experiment was repeated three times. **c** and **d**. Downregulation of SRC decreased cell invasion and migration ability. The images shown are representative images from three independent experiments, and a statistical analysis was performed (mean ± SE; *** p <0.001;****P <0.0001) vs. control group.

To investigate whether the regulatory effects of miR-940 on the proliferation, invasion and migration of OC cells are mediated by SRC, we applied siRNA-mediated SRC inhibition method to analyze whether it could replicated the tumor suppressor of miR-940 in OC cell lines. SiRNA-mediated suppression of SRC and its downstream genes was confirmed by Western blot analysis (Figure 8a). As Expected, SRC knockdown significantly inhibited colony formation ability (Figure 8b). Similarly, SRC knockdown suppressed cell invasion and migration of OC cells (Figure 8c, 8d, respectively). These results were similar to the effects of miR-940 overexpression.

### Neutral sphingomyelinase 2 inhibitor (GW4869) reduces exosome secretion and exocytosis of miR-940

**Figure 9:**
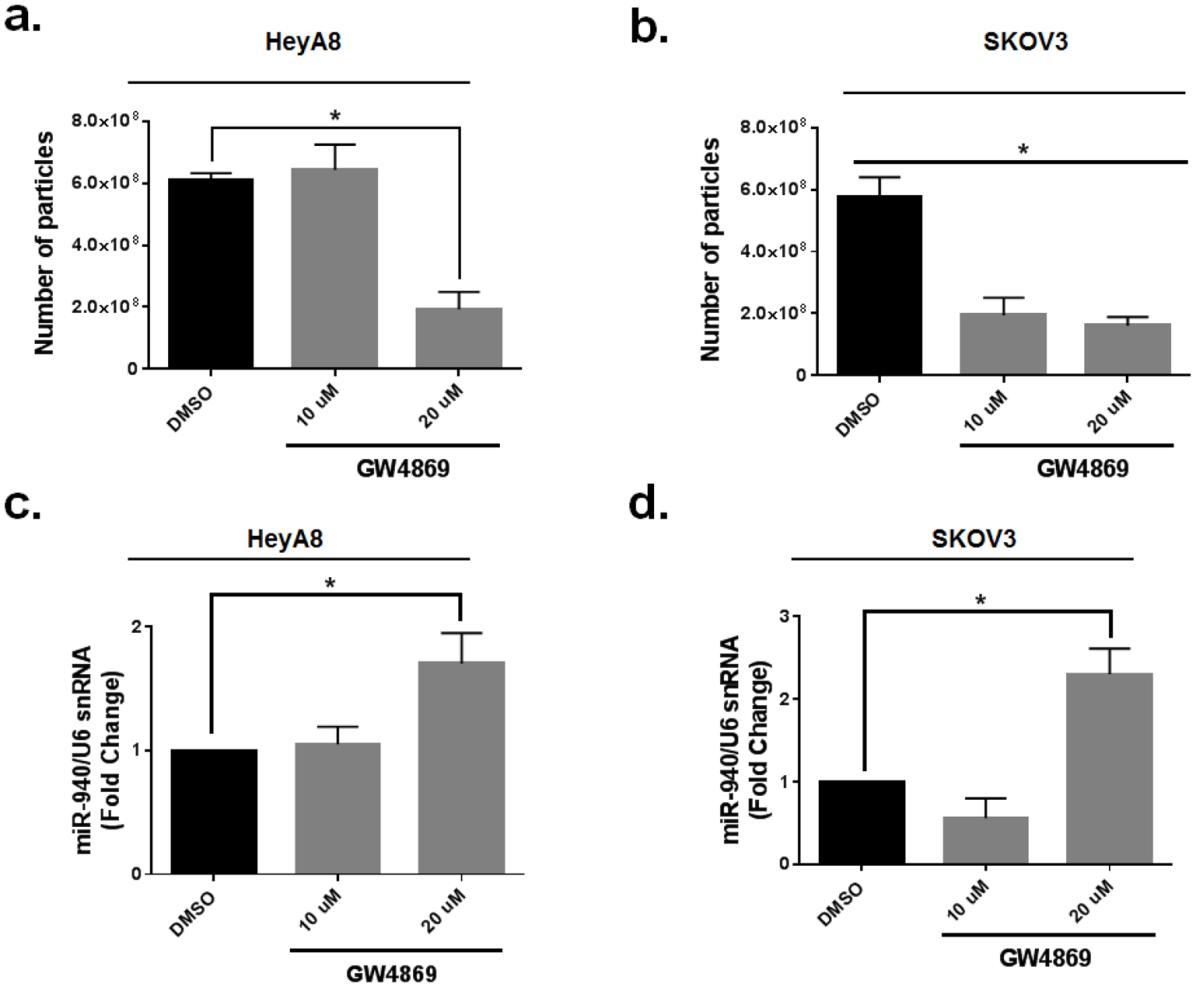
Neutral sphingomyelinase 2 inhibitor (GW4869) reduces exosomal miR-940 secretion in OC cells **a** and **b**. Quantification of exosomes by Nanoparticle Tracking Analysis. Data are presented as mean± SE. *, p <0.05. (Student t-test). The experiment was repeated two times. **c** and **d**. Inhibition of nSMase caused a significant increase in intracellular levels of miR-940 in in treated groups. The data are shown as mean±. SE of two separate experiments. *P <0.051indicates significant difference compared with vehicle control groups.

Emerging evidence suggests that the secretion of miRNAs in exosomes is dependent on ceramide via its production by neutral sphingomyelinase 2 (nSMase 2) [43, 44]. Inhibition of de novo ceramide synthesis by treatment with an nSMase inhibitor impaired exosomal miRNA release, apparently due to decreased formation of miRNA-containing exosomes [45, 46]. To determine the effect of nSMase inhibitor, GW4869 on exosomal miR-940 secretion in OC cells, we treated both HeyA8 and SKOV3IP1 cell lines with GW4869 or dimethylsulfoxide vehicle for 24 h. GW4869 treatment significantly reduced the number of vesicles present in exosomes as detected by NTA (Figure 9a, 9b). Inhibition of nSMase caused a ~ threefold increase in intracellular levels of miR-940 in in treated groups than vehicle control groups (Figure 9c, 9d).

Furthermore, treatment of ovarian cancer cells with GW4886 led to decrease in cancer cell invasion and migration (Supplementary Figure 7a and 7b). We also checked p-SRC levels by western blotting, as well as major apoptosis markers. p-SRC levels, as well as major apoptosis markers such as pro-caspase 3 and 9 were dramatically decreased by GW4886 treatment (Supplementary Figure 7c).

## DISCUSSION

In the present study, we found that miR-940 is highly expressed in exosomes derived from cancer cells, while not in exosomes from normal cells. Thus, the incorporation of miR-940 in exosomes may represent a characteristic of cancer cells not found in normal cells. We also found that ectopic expression of miR-940 resulted in antitumor activity and decreased tumor cell proliferation *in vivo* and *in vitro*. This findings may suggest an exclusionary mechanism used by cancer cells to down regulate tumor suppressor miRNAs. A previous study showed that the let-7 miRNA family is abundant in two OC cell exosomes and suggested that the release of exosomes and miRNA content of exosomes significantly differ between OC cell lines and correlate with their invasive potential [47]. In our recent study, we also showed that both resistant and sensitive ovarian cancer cells release miR-6126; a tumor suppressor targeting integrin beta-1 via their exosomes [48]. Ohshima and colleagues suggested that metastatic gastric cancer cells release let-7 miRNAs into the extracellular environment via exosomes to maintain their oncogenesis [49]. Another recent study showed that metastatic cells from bladder carcinoma discard via exosomes the tumor suppressor miRNAs that restrict their metastatic progression [50]. All these observations are in line with the data obtained in our study.

Exosomal miRNAs also function as mediators of cell-cell communication, which may lead to cancer cell growth, migration, invasion, dissemination, and metastasis or the impairment of the immune system response [51–53]. Our findings indicate that cancer cells, use exosomes for intercellular communication and to discard tumor suppressor miRNAs to maintain an endogenous balance between tumor suppressor miRNAs and their oncogenic targets.

To date, several studies described the role of miR-940 in different types of cancers including breast cancer [54], hepatocellular carcinoma [55], nasopharyngeal carcinoma [56], pancreatic ductal adenocarcinoma [57], and prostate cancer [58]. It was reported in many studies that miR-940 was highly expressed in normal tissues compared with tumors, and miR-940 was shown to inhibit the migratory and invasive potential of cancer cells by suppressing CXCR2 [59], MIEN1 [58], MyD88 [57], Nestin [56], and ZNF24 [54]. Moreover, the Estrogen-Related Receptor Gamma (ESRRG) was targeted by miR-940, and suppression of ESRRG inhibited hepatocellular carcinoma cell lines growth and induced cell apoptosis. The knockdown of miR-940 restored MKP1 and MKK4 expression and attenuated cisplatin resistance in lung cancer. Paradoxically, miR-940 has been demonstrated to promote tumor cell invasion and metastasis by downregulating GSK3β/sFRP1 [60] and ZNF24 in gastric cancer [61], respectively. However, the clinical significance of miR-940 and the biological roles of miR-940 and its direct functional targets in OC has not yet been evaluated. A key observation in this study is the regulation of SRC by miR-940 administration, exerting a tumor suppressor effect in both *in vivo* and *in vitro*. The SRC proto-oncogene is a non-receptor tyrosine kinase that is involved in a variety of cellular functions including cell proliferation, invasion, migration, and apoptosis [62]. Src kinases have been reported to be overexpressed and activated in a high proportion of ovarian cancers [63].

Inhibition of neutral sphingomyelinase 2 (nSMase 2), either by small inhibitor or siRNA have been found previously to regulate secretion of exosomes [43, 64, 65]. In the present study, we used GW4869, which is an nSMase2 inhibitor, to confirm that intracellular miR-940 down-regulation is an exosome-dependent process. Results showed that miR-940 accumulated in cells following the inhibition of exosome membrane formation by GW4869.

Several studies have concentrated on miRNA signatures of exosomes derived from cancer cells such as ovarian, breast, pancreatic, prostate, and others [18, 66–69], but few have assessed the function of these miRNAs. Furthermore, exploration of the effects of the released miRNAs on the microenvironment of various types of cancer cells may elucidate potential homeostatic regulation at this level.

Taken together, our findings shed light on how OC cells use exosomal pathways as a mechanism whereby cells rapidly discard specific tumor-suppressor conforming the hypothesis that malignant cells may release their tumor-suppressor miRNAs via exosomes into the extracellular environment to maintain and promote tumorigenesis at the intracellular level. The release of miR-940-rich exosomes may represent a new and additional layer of regulation of miR-940 expression levels and, in turn, of the SRC mRNA that is targeted by this miRNA and critically control OC progression.

## MATERIALS AND METHODS

### Cell lines and culture conditions

The human epithelial OC cell lines were obtained from American Type Culture Collection (Manassas, VA). HeyA8, SKOV3IP1, A2780-PAR, and A2780-CP20 and the non-transformed human ovarian surface epithelial cell line HIO-180 were maintained as described previously [70–72]. The taxane-resistant cell lines HeyA8-MDR and SKOV3-TR were maintained in Roswell Park Memorial Institute 1640 (RPMI 1640) medium (Life Technologies) supplemented with 15% fetal bovine serum (FBS) and 1% penicillin/streptomycin with paclitaxel (300 ng ml^−1^ for HeyA8-MDR; 150 ng ml^−1^ for SKOV3-TR). The A2780-CP20 cell line was developed by sequential exposure of the A2780 cell line to increasing concentrations of cisplatin. HEK293 (human embryonic kidney) cells were maintained in Dulbecco's modified Eagle medium (Life Technologies) supplemented with 10% FBS and 1% penicillin/streptomycin. In addition, cell lines were routinely tested to confirm the absence of *Mycoplasma*, and all experiments were performed with cultures at 60-70% confluence.

### Exosome isolation from cell media

Exosomes are traditionally isolated from conditioned media by serial centrifugation at a low speed, followed by ultracentrifugation at 100,000 × *g* to pellet the exosomes [73, 74]. Recently, a proprietary method of exosome isolation was made commercially available and was reported to provide a rapid and efficient method for exosome isolation [23, 75, 76]. Briefly, cells were cultured for 48 hours; thereafter, media were replaced with RPMI 1640 supplemented with 10% exosome-depleted FBS [77]. (EXO-FBS-250 A-1, System Biosciences). After 24 hours, cells and debris were removed by centrifugation at 350 *g* for 10 minutes and 2000 *g* for 30 minutes. This was followed by filtration of the supernatant with 0.2 μm filters. Next, each sample was combined with ½^th^ volume of total exosome isolation reagent (Invitrogen, Life Technologies). Cell medium and the exosome isolation reagent were mixed by brief vortexing until a homogeneous solution was formed and incubated at 4°C overnight before centrifugation at 4°C at 10,000 *g* for 1 hour. The supernatant was aspirated and discarded, and the exosome pellet was resuspended in phosphate-buffered saline (PBS) and stored at −20°C until further use.

### Atomic force microscopy

For atomic force microscopy (AFM) imaging of isolated exosomes, samples were diluted 1:100 in deionized water and adsorbed to freshly cleaved mica sheets for 10 minutes. The sheets were rinsed thoroughly with deionized water to remove unbound exosomes and dried under a gentle stream of nitrogen. A Bioscope II (Veeco Digital Instruments, Santa Barbara, CA) was used for tapping mode AFM imaging using silicon probes with spring constant *k* = 305 KHz (OTESPA, Veeco). Topographic height and phase images were recorded simultaneously at 512×512 pixels at a scan rate of 0.4 Hz. The height of the exosomes was obtained from a line profile of height images (Nanoscope software). Image processing was performed using the free WSxM software (Nanotec, Spain).

### Nanoparticle tracking analysis

Isolated exosomes were suspended in 200 μl of PBS. We obtained 5 μl of this sample, diluted it 1:100 in 1x PBS, and analyzed it using a NanoSight LM10 instrument, following the manufacturer's protocol (NanoSight Ltd., Amesbury, UK). Exosomes were illuminated by the laser, and their movement under Brownian motion was recorded in videos. The nanoparticle tracking analysis (NTA) software uses the video data for tracking the Brownian motion of individual vesicles and thereby calculating their size and total concentration. All samples were evaluated in triplicate.

### Western blot analysis

Cells and exosomes were lysed in1X RIPA buffer (Thermo Scientific) containing protease and phosphatase inhibitors. Lysates were centrifuged, supernatants were collected, and the total protein concentration in lysates was assessed using the BCA protein assay kit (Pierce Biotechnology). Subsequently, lysates were resuspended in Laemmli loading buffer (Bio-Rad) and heated at 95°C for 5 minutes. Next, Western blotting was performed as previously reported [78, 79]. All antibodies used in this study are listed in Supplementary Table 1.

### RNA isolation

Cellular and exosomal RNA were isolated using the miRCURY RNA Isolation Kit (Exiqon) according to the manufacturer's protocol and as described previously [80, 81]. Eldh and colleagues demonstrated that, for both cells and exosomes, the miRCURY pure column-based method showed the highest total RNA yield and the broadest RNA size distribution when compared with 6 other RNA isolation methods [82]. The quantity and quality of the RNA extractions were determined using an Agilent 2100 Bioanalyzer with the RNA 6000 Nano or RNA 6000 Pico Kit according to the manufacturer's protocol (Agilent Technologies).

### miRNA microarray expression analysis

For miRNA expression analysis, we used a GeneChip 4.0 miRNA microarray (Affymetrix), which covers all of the 2,578 mature human miRNAs available in miRBase version 20 (www.mirbase.org/). Hybridization and scanning of the samples were performed by the Noncoding RNA Program at MD Anderson's Center for Targeted Therapy. Briefly, 600 ng of total RNA of each sample was subjected to a tailing reaction labeled with the FlashTag Biotin HSR RNA Labeling Kit (Affymetrix) followed by ligation of the biotinylated signal molecule to the target RNA sample according to the manufacturer's instructions. Each sample was then hybridized to a GeneChip 4.0 miRNA array at 48°C for 16 hours in the GeneChip Hybridization Oven 645 and rotated at 60 rpm. Immediately following hybridization, the probe array underwent an automated washing and staining protocol on the GeneChip Fluidics Station 450. Once the probe array was hybridized, washed, and stained, it was scanned using a GeneChip Scanner 3000 7G. The expression values were summarized and normalized with the robust multichip analysis program Affymetrix Expression Console 1.3. AffymetrixTranscriptome Analysis Console software was used for additional statistical analysis. Differentially expressed miRNAs were identified by using one-way analysis of variance (ANOVA) with p- values cutoff by 0.05 and fold change more than 2.0 or less than -2.

### Quantitative real-time polymerase chain reaction

For mRNA quantification, cDNA was synthesized from 1 μg of isolated RNA using a QuantiTect Reverse Transcription Kit (Qiagen) according to the manufacturer's instructions. Quantitative real-time polymerase chain reaction (qRT-PCR) was carried out with BX-384 Bio-Rad using the QuantiTect SYBR Green PCR kit (Qiagen) according to the manufacturer's protocol. To quantify miRNAs, 1 μg of total RNA was reverse transcribed with a qScript miRNA cDNA Synthesis Kit (Quanta Biosciences), and qRT-PCR was carried out using PerfeCta SYBR Green SuperMix (Quanta Biosciences). All reactions were performed in triplicate. Relative quantification of mRNA and miRNA expression was calculated with the 2−ΔΔCt method. Transcript values were normalized to those obtained from the amplification of the internal control (glyceraldehyde 3-phosphate dehydrogenase) for mRNAs and U6 snRNA for intracellular and extracellular miRNAs. All miRNA Assay Primers used in this study were purchased commercially (Quanta Biosciences or Qiagen), and are based on human miRNA and snRNA sequences.

### *In vitro* miRNA and siRNA transfection

Cells were seeded at 1 × 10^5^ per well in 6-well plates and allowed to attach for at least 16 hours. The mirvana miRNA mimic of microRNA-940 mimic (miR-940) or small interfering RNA (siRNA) targeting SRC, and their negative controls were transfected using HiPerFect transfection reagent (Qiagen) (at a final concentration of 50 nM) for 72 hours. Media were changed 6 hours following transfections to minimize toxicity. All the siRNAs were obtained from Sigma and miRNAs were obtained from Life Technologies. All the oligonucleotide sequences are listed in Supplementary Table 2. All the siRNAs were obtained from Sigma and miRNAs were obtained from Life Technologies. MiRVana miRNA mimic negative control #1 (catalogue number 4464061, ThermoFisher Scientific) was used as the negative control.

### Cell viability and proliferation assays

The viability of cells was detected by 3-(4,5-dimethylthiazol-2-yl)-5-(3-carboxy-methoxyphenyl)-2-(4-sulfophenyl)-2H-tetrazolium (MTS) assay (Promega). Viable cells were seeded at a density of 1000 cells/well in 96-well plates and transfected with 50 nM control or miR-940 mimics for 48 hours. After treatment, a solution containing MTS and phenazinemethosulfate (20:1 v/v) was added to the cells for 1-3 hours at 37°C, and the viable growing cells were estimated by monitoring the absorption of the product at 490 nm, based on the generation of formazan in live cells. All experiments were performed in 6 replicates, and the results were reported as the mean absorption ± standard deviation.

### Matrigel invasion assay

The invasiveness of HeyA8, HeyA8-MDR, and SKOV3IP1 cells was determined as previously described [83]. Briefly, cells were treated with 50 nM of the indicated oligonucleotide, and 48 hours later, live cells were collected and washed with serum-free media. Fixed numbers of the viable cells (4 × 10^5^ cells), were resuspended in 1 mL of serum-free RPMI-1640 medium and added onto 6-well plate Transwell inserts (8-μm-pore size; Fisher Scientific) coated with a Matrigel basement membrane (0.7 mg/mL; BD Biosciences). Lower chambers were filled with 2 mL of medium supplemented with 15% FBS. After 24 hours, non-invading cells on the upper surface of the filter were removed with cotton swabs. Cells that invaded through the Matrigel onto the lower side of the filter were fixed, stained with the Hema-3 Stain System (Fisher Scientific), and photographed. The number of cells that invaded the lower side of the filter was counted in 5 fields and expressed as the mean number of cells from triplicate measurements.

### Wound-healing assay

Ovarian cancer cells were seeded in 6-well plates (1 × 10^5^cells) and cultured to form a confluent monolayer. Cells were transfected with 50 nM of the indicated oligonucleotide for 48 hours. Wounds were then carefully made on the cell layer using 10 μl sterile micropipette tips, and cells were washed several times with appropriate culture medium to remove cell debris. Immediately after scratches were made, the cells were photographed using a phase-contrast microscope (Nikon Instruments Inc.), to determine the wound width at 0 hours. The cultures were continued, and the cells were photographed again after 12 hours and after 24 hours of wounding the cell layer. The wound healing was visualized by comparing photographs taken at 0 hours with those taken 12 and 24 hours later. At least 5 random non-overlapping images per experiment were analyzed and quantitated using Image J software (National Institutes of Health, Bethesda, MD). Three experiments were done in duplicate.

### Colony formation assay

Cells were seeded in 6-well plates at a low density (1000 cells/plate), transfected with miRNA or siRNA and their respective negative controls, and allowed to grow until visible colonies appeared. Then, the formed colonies were stained with crystal violet and counted. Each experiment was performed in duplicate.

### Flow cytometry analysis of cell-cycle

Cells were transfected as previously described with miRNA mimics. Samples were washed in PBS and fixed in 75% ethanol overnight. Cells were then centrifuged and reconstituted in PBS containing 50 μg/mL propidium iodide (PI) and 100 U/mL of RNAse A. Cells were incubated at 37°C for 30 minutes in the dark before flow cytometry analysis. CellQuest Pro software (BD Biosciences) was used to determine the number of cells in each phase of the cell cycle.

### Apoptosis

Cell apoptosis was assessed by Annexin V/PI staining using an FITC apoptosis detection kit (BD Biosciences) according to the manufacturer's protocol. Apoptotic cells were analyzed with a FACSCalibur flow cytometer (BD Biosciences). CellQuest Pro software (BD Biosciences) was used to determine the number of apoptotic cells. Apoptosis was also assessed by monitoring the cleavage of caspase-3, caspase-9, and poly ADP ribose polymerase (PARP) via Western blotting.

### Luciferase reporter assay

Luciferase reporter plasmids containing fragments of the wild-type 3′UTR (CS-HmiT064432-MT06-02 and CS-HmiT064432-MT06-03) and the mutated 3′UTR (CS-HmiT064432-MT06-02a and CS-HmiT064432-MT06-03a) miR-940 binding site of SRC (NM_198291) were specifically obtained from Genecopoeia (Rockville, MD). Luciferase activity assays were performed following the manufacturer's protocols. Briefly, HEK293 cells were seeded in 6-well plates and co-transfected with the luciferase constructs and miR-940 or miR-Control using Lipofectamin 2000 (Invitrogen). The cells were transferred to a 96-well plate 24 hours after transfection and cultured for another 24 hours. Luciferase activity was measured using a Dual-Luciferase Reporter Assay according to the manufacturer's instructions (Genecopoeia). Results represent three independent experiments, and each was performed in triplicate.

### Inhibition of exosome release

To validate whether miR-940 disposal depends on exosome transfer, exosome release was blocked using GW4869 (D1692; Sigma-Aldrich, USA), which is a specific chemical inhibitor for nSMase2. HeyA8 and SKOV3IP1 cells were pre-seeded in a T25 flask and cultured for 24 h in complete medium. After the incubation, the medium was switched to fresh exosome free medium with different GW4869 concentrations. The cells were collected, and exosome fraction was obtained from the culture medium after 24-h incubation.

### Orthotopic *in vivo* model

To generate tumors, 2.5×10^5^ HeyA8 cells were injected into the peritoneal cavity of 8- to 12-week-old female athymic nude mice (Frederick Cancer Research and Development Center, Frederick, MD). The mice were cared for in accordance with guidelines set forth by the American Association for Accreditation of Laboratory Animal Care and the United States Public Health Service Policy on Human Care and Use of Laboratory Animals. All mouse studies were approved and supervised by the MD Anderson Institutional Animal Care and Use Committee. One week after tumor cell injection, mice were randomly assigned to receive miR-control or miR-940 intravenously (10 mice per group). All miRNAs for *in vivo* delivery were incorporated into 1, 2-dioleoyl-*sn*-glycero-3-phosphatidylcholine (DOPC) nanoliposomes as previously described [3]. All groups received twice-weekly miRNA treatments for 4 weeks. When treatment was completed, the mice were euthanized. After euthanasia, mouse and tumor weight, the number of nodules, and the distribution of tumors were recorded. Tumor tissues were fixed in formalin and embedded in paraffin or were snap-frozen in liquid nitrogen for immunohistochemical analysis.

### Immunohistochemical analysis

The immunohistochemical analysis for tumors cell proliferation was performed as previously described [79, 83, 84]. The antibodies used and the vendors are listed in Supplementary Table 1.

### Survival analysis

We downloaded miRNA-Seq Level3 data publicly available from the Cancer Genome Atlas Project (TCGA; http://tcga-data.nci.nih.gov/ additional file 5) for miR-940 in patients with ovarian serous cystadenocarcinoma (OV). For the miRNA-Seq data, we derived the ‘reads_per_miallion_miRNA_mapped’ values for the mature form MIMAT0004983 from the ‘isoform_quantification’ files. Patient overall survival information was retrieved from cbioPortal (http://www.cbioportal.org/). The analyses were carried out in R statistical environment (version 3.0.1) (http:/// www.r-project.org/). All the tests were two-sided and considered statistically significant at the 0.05 level. For miR-940, the tumor samples were dichotomized into high and low miRNA expression groups at percentile cutoffs between 0.25 and 0.75 with a step of 0.01. We tested whether a log-rank test applied at any cut-point would yield a nominal P value <0.05. The optimal cutoff percentile (as determined by the lowest P value) was identified for miR-940 as 0.58 (range for low expression =0.689-4.339, and for high expression =4.343-7.317 (log2). The Kaplan-Meier plots were generated for high and low miR-940. The numbers of patients at risk in each group at different time points are presented at the bottom of the graph. A table with patients overall survival and miRNA expression is provided in the supplementary material.

### Statistical analysis

Unless specified otherwise, the data are expressed as the mean ± standard error of three independent experiments. Statistical analysis was performed using the Student's *t* test to determine statistical significance, and *P* values indicate the probability of the means compared, being equal with *P <0.05, **P <0.01, ***P <0.001, and ****P <0.0001. Student's *t*-tests and ANOVA were calculated with GraphPad software.

## SUPPLEMENTARY MATERIALS FIGURES AND TABLES












