# Supplementary material for: Exosomal miR-940 maintains SRC-mediated oncogenic activity in cancer cells: a possible role for exosomal disposal of tumor suppressor miRNAs
Source: Oncotarget. 2017 Feb 20;8(12):20145–64. doi: 10.18632/oncotarget.15525 (PMC5386751; doi:10.18632/oncotarget.15525)
Supplement: Supplementary file 1 [file oncotarget-08-20145-s001.pdf]

Exosomal miR-940 maintains SRC-mediated oncogenic activity in cancer cells: a possible role for exosomal disposal of tumor suppressor miRNAs

SUPPLEMENTARY DATA

SUPPLEMENTARY FIGURES AND TABLES

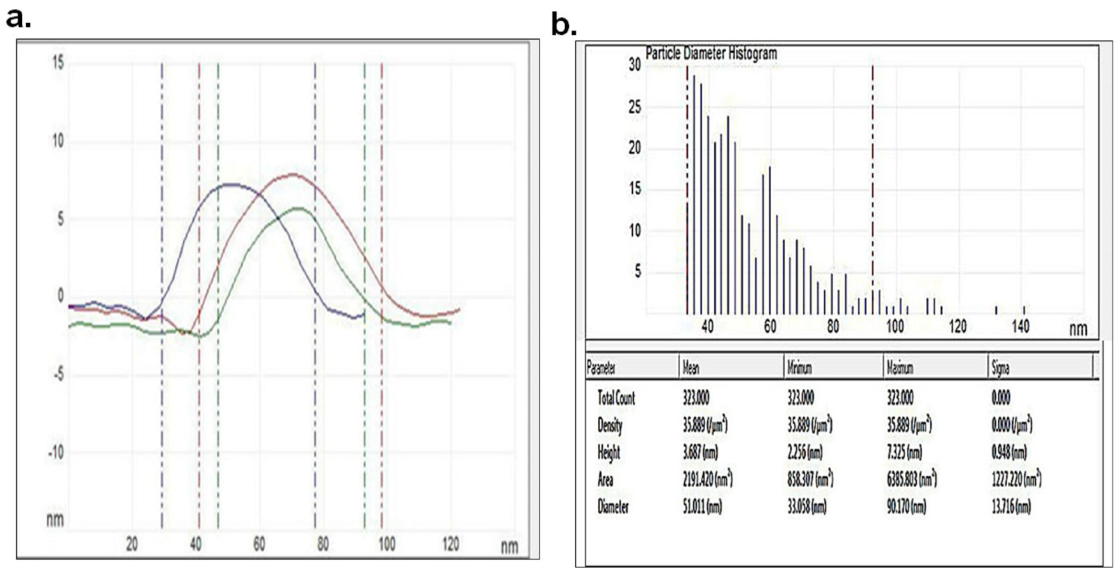

Supplementary Figure 1: Atomic force microscopy images of exosome. a. Graphical representation of the size distribution of exosomes showing near homogeneity with respect to height and width. **b.** Exosomes size distribution, determined on the basis of AFM images of 323 vesicles, is shown by histograms.

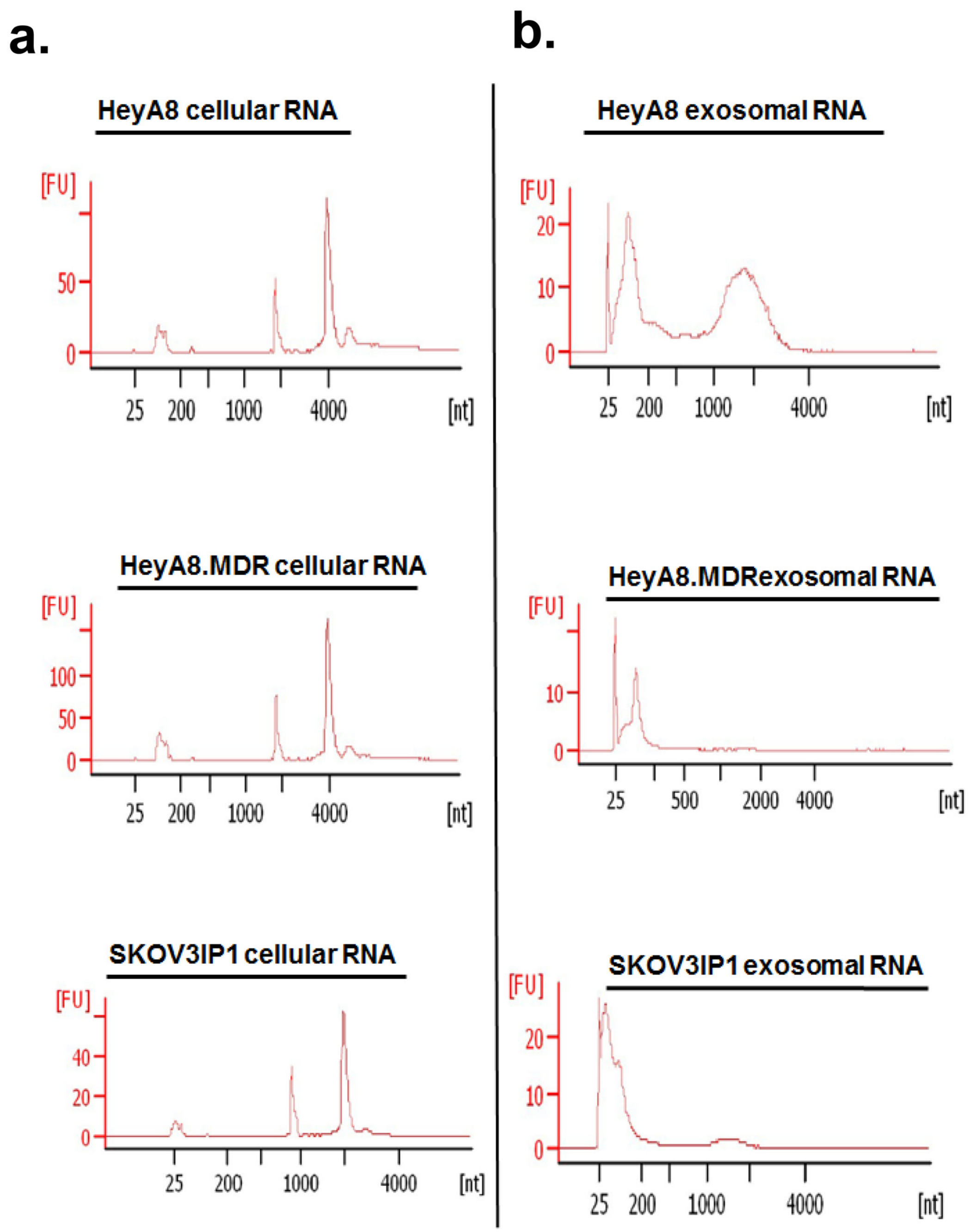

Supplementary Figure 2: Exosomal and cellular RNAs analyzed using a Bioanalyzer 2100. Total RNA isolated from exosomes **b.** is enriched in small RNAs and contains no or very low amounts of ribosomal RNA (18S and 28S rRNAs) compared to total RNA from cells **a.** The peak at around 25 nucleotides represents an internal standard.

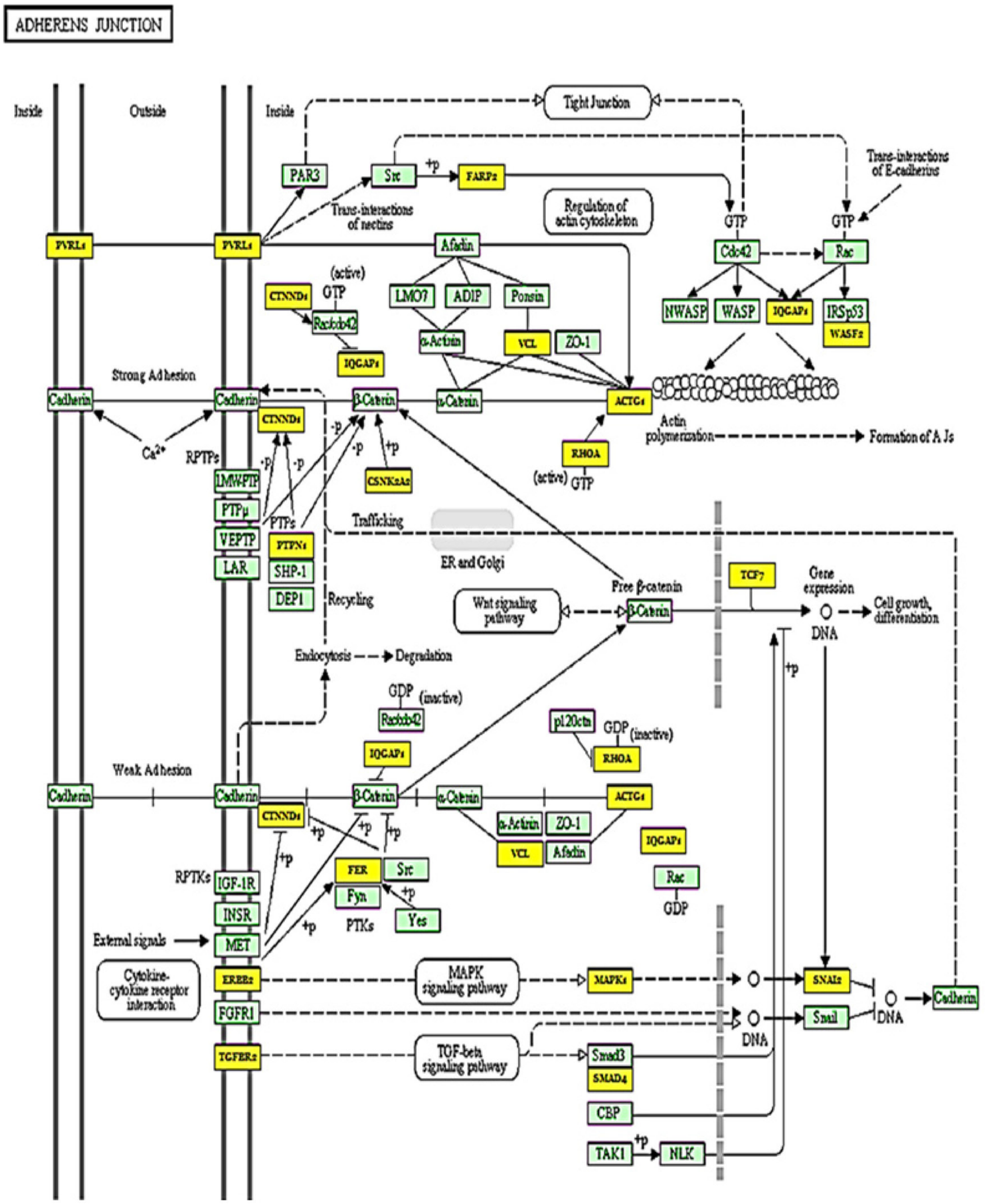

Supplementary Figure 3: Targets of selectively exported miRNAs are involved in adherence junction pathways. Adherence junction pathway (Kegg: hsa: 04520) is depicted with the target genes of the 4 most exported miRNAs. Those genes predicted by Diana microT-CDS are highlighted in yellow.

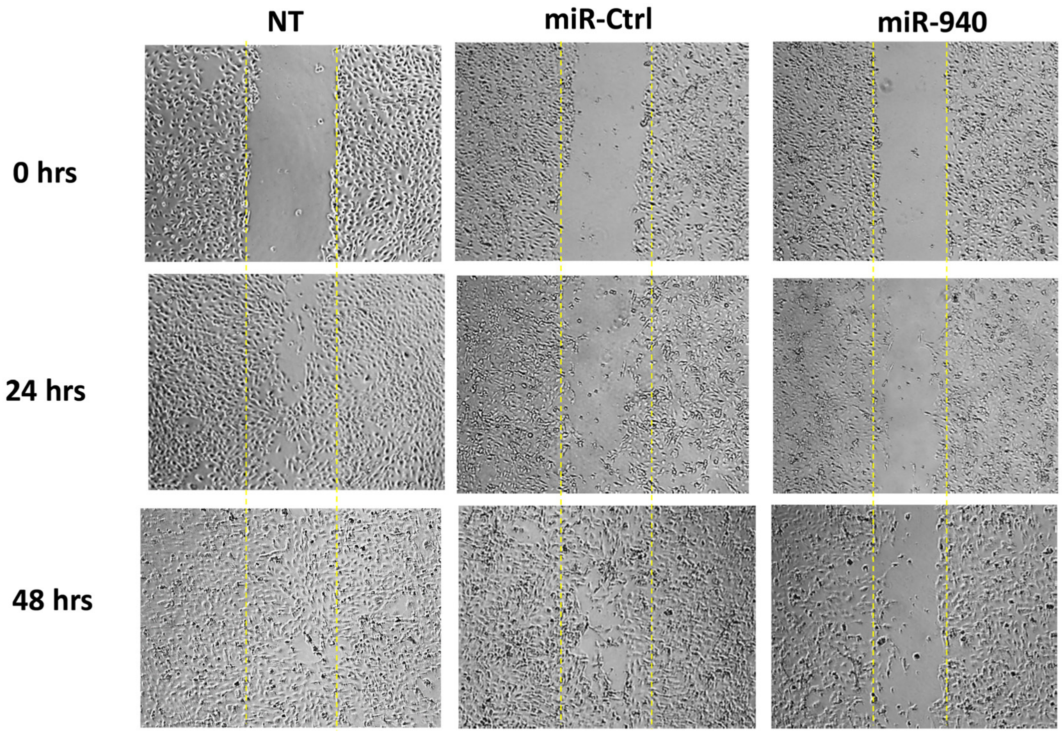

Supplementary Figure 4: The effect of miR-940 overexpression in normal HIO-180 cells. HIO-180 cells were transfected with CTL miRNA mimic or miR-940 mimic. Pictures were taken after 24h and 48h following scratch formation (t=0). Wound healing assay showed that the migration of HIO-180 ovarian cells was decreased upon miR-940 mimic transfection compared to control groups.

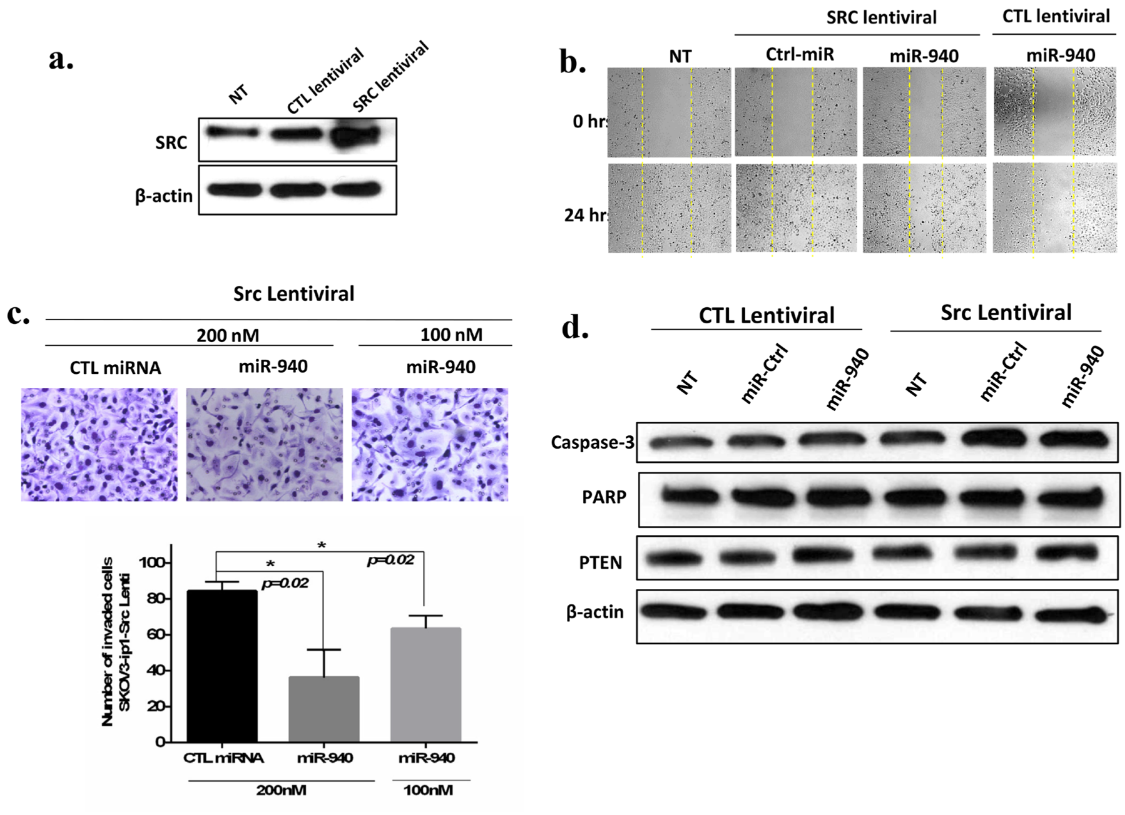

Supplementary Figure 5: Ectopic expression of miR-940 rescued the increased invasive and migratory effect of SRC in ovarian cancer cells. a. SRC levels were increased upon SRC lentiviral transfection in ovarian cancer cell. **b.** Treatment of SRC lentiviral transfected cells with miR-940 resulted in smaller open wound area compared to CTL miRNA treated cells. **c.** miR-940 treatment decrease the invasion compared to CTL miRNA treated cells in Src lentiviral transfected cells. **d**. miR-940 treatment did not alter the expression of apoptotic proteins such as PART and Caspase-3.

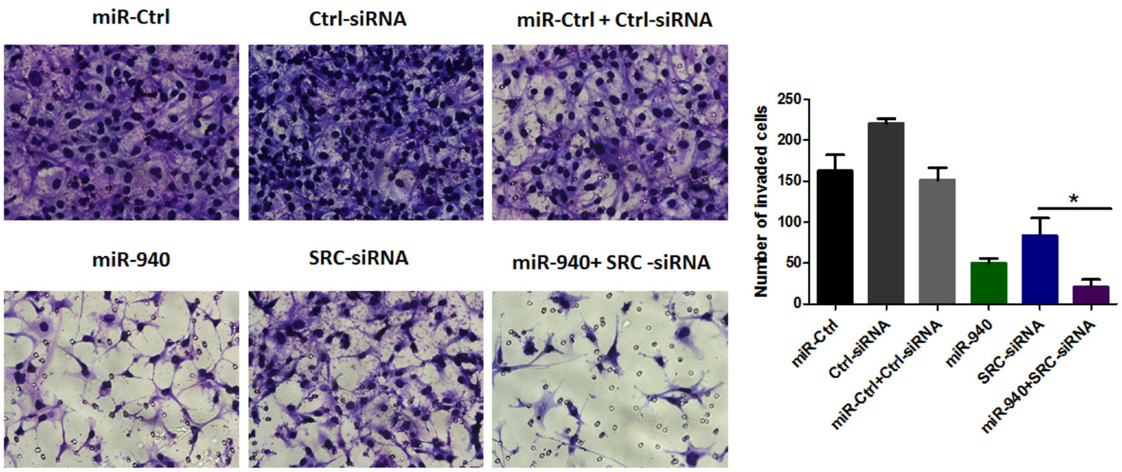

Supplementary Figure 6: miR-940 has synergistic effect on ovarian cancer cell invasion *in vitro*. miR-940 treatment and SRC siRNA treatment resulted in decrease in cancer cell invasion. Combination treatment resulted in synergestic effect by a significant decrease in number of invaded cells compared to SRC siRNA alone.

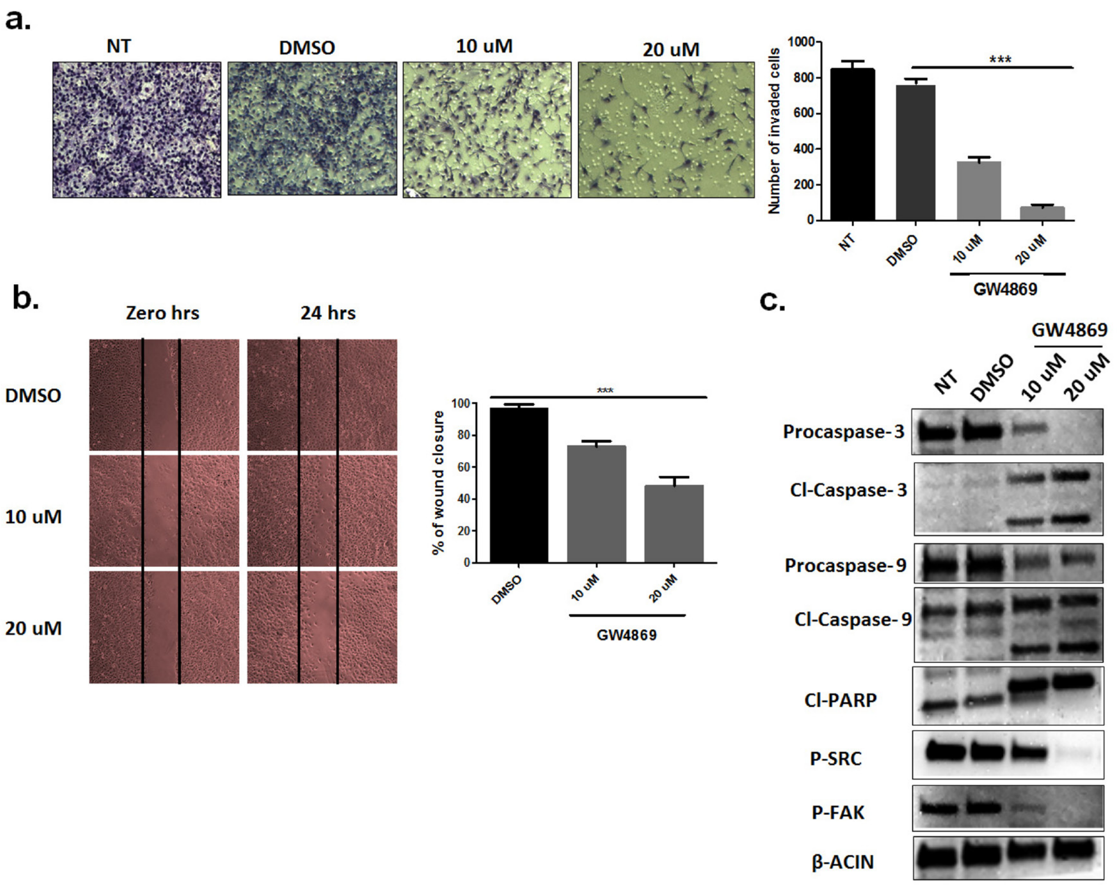

Supplementary Figure 7: GW4886 inhibits cancer cell metastasis *in vitro*. Ovarian cancer cells treated with 10uM and 20 uM GW4886 treatment resulted in decreased cancer cell **a.** migration and **b.** invasion. **c.** Apoptotic markers such as Procaspase-3 and pro-caspase-9 were decreased by GW4886 treatment in ovarian cancer cells.

Supplementary Table 1: Antibodies used in the Western blotting and immunohistochemical analyses

Target Protein	Source	Cat #	Applications	Dilution
CD63	System Biosciences (SBI)	EXOAB-KIT-1	WB	1:1000
CD9	System Biosciences (SBI	EXOAB-KIT-1	WB	1:1000
HSP70	System Biosciences (SBI	EXOAB-KIT-1	WB	1:1000
Cytochrome c	Cell Signaling Technology	11940	WB	1:1000
Vinculin	Santa Cruz Biotechnology	sc-7649	WB	1:200
PARP	Cell Signaling Technology	9542	WB	1:1000
Caspase-3	Cell Signaling Technology	9662	WB	1:1000
Cleaved Caspase-3 (Asp175)	Cell Signaling Technology	9661	WB	1:1000
Caspase-9	Cell Signaling Technology	9502	WB	1:1000
Cleaved Caspase-9 (Asp330)	Cell Signaling Technology	9501	WB	1:1000
Survivin	Cell Signaling Technology	2808	WB	1:1000
PTEN	Cell Signaling Technology	9559	WB	1:1000
Actin-beta	Sigma-Aldrich	A5316	WB	1:3500
GAPDH	Santa Cruz Biotechnology	Sc-25778	WB	1:200
Src	Cell Signaling Technology	2123	WB	1:1000
Src (p416)	Cell Signaling Technology	6943	WB	1:1000
FAK (pY397)	BD Biosciences	611722	WB	1:1000
FAK	BD Biosciences	610087	WB	1:1000
Akt (pS473)	Cell Signaling Technology	4060S	WB	1:1000
Akt	Cell Signaling Technology	9272	WB	1:1000
Paxillin (pY118)	Cell Signaling Technology	2541	WB	1:1000
Paxillin	Cell Signaling Technology	2542	WB	1:1000
c-Jun	Santa Cruz Biotechnology	sc-1694	WB	1:200
Ki67	Thermo Scientific	RB9043-P	IHC	1:300

Abbreviations: WB, western blot; IHC, immunochemistry; FAK, focal adhesion kinase; PARP, poly ADP ribose polymerase; GAPDH, Glyceraldehyde-3-phosphate dehydrogenase

Supplementary Table 2: List of oligonucleotide sequences used in this study

Name	Target Sequences	Cat #
**Control siRNA (Ctl siRNA)**	5′-UUCUCCGAACGUGUCACGUUU-3′	WD00909801
**SRC-siRNA**	5′-CAGUUGUAUGCUGUGGUUU -3′	SASI_HS01_00112907
**hsa-miR-940 mimic (miR-940)**	AAGGCAGGGCCCCCGCUCCCC	MC12798

Additional File 1: The list of top most abundant miRNAs in exosomes versus their cells of origin

See Additional File 1

Additional File 2: The list of top most enriched miRNAs in OC-derived exosomes versus normal exosomes

See Additional File 2

Additional File 3: The genes list predicted to be targeted by one or more of the most selectively exported miRNAs

See Additional File 3

Additional File 4: Predicted pathways targeted by miRNAs highly enriched in OC-derived exosomes

See Additional File 4

Additional File 5: TCGA data patient expression values

See Additional File 5
